# Degraded Psychophysiological Status in Caregivers and Human Resources Staff during a COVID-19 Peak Unveiled by Psychological and HRV Testing at Workplace

**DOI:** 10.3390/ijerph19031710

**Published:** 2022-02-02

**Authors:** Véronique Deschodt-Arsac, Valérie Berger, Leila Khlouf, Laurent M. Arsac

**Affiliations:** 1 Univ. Bordeaux, CNRS, Laboratoire IMS, UMR 5218, 33400 Talence, France; valerie.berger@chu-bordeaux.fr (V.B.); leilakhlouf13@hotmail.com (L.K.); laurent.arsac@u-bordeaux.fr (L.M.A.); 2 CHU de Bordeaux, 33000 Bordeaux, France

**Keywords:** COVID-19, caregivers in frontline, heart rate variability, multiscale entropy, anxiety, coping strategies, multivariate analyses

## Abstract

During COVID-19 pandemic peaks, healthcare professionals are a frontline workforce that deals with death on an almost daily basis and experiences a marked increase in workload. Returning home is also associated with fear of contaminating or be contaminated. An obvious consequence is stress accumulation and associated risks, especially in caregivers in mobility and possibly in human resource teams managing mobility. Here, during the second pandemic peak, we designed a 15-min testing procedure at the workplace, combining HADS and Brief COPE questionnaires with heart rate variability (HRV) recordings to evaluate psychophysiological status in four groups: caregivers in mobility (MOB); human resources teams managing mobility (ADM); caregivers without mobility (N-MOB); and university researchers teaching online (RES). Anxiety, depression, coping strategies, vagally-mediated heart rate regulation, and nonlinear dynamics (entropy) in cardiac autonomic control were quantified. Anxiety reached remarkably high levels in both MOB and ADM, which was reflected in vagal and nonlinear HRV markers. ADM maintained a better problem-solving capacity. MOB and N-MOB exhibited degraded problem-solving capacity. Multivariate approaches show how combining psychological and physiological markers helps draw highly group-specific psychophysiological profiles. Entropy in HRV and problem-solving capacity were highly relevant for that. Combining HADS and Brief COPE questionnaires with HRV testing at the workplace may provide highly relevant cues to manage mobility during crises as well as prevent health risks, absenteeism, and more generally malfunction incidents at hospitals.

## 1. Introduction

The coronavirus disease 2019 (COVID-19) pandemic has affected the working conditions of a number of professions. Specifically in the health sector, disruptions have reached an unprecedented degree due to the severity and duration of the crisis. This real upheaval can have serious consequences for health in caregivers, especially in emergency and resuscitation teams during the pandemic peaks where overwork is obvious but not the unique risk factor [1]. Although in these departments, caregivers are on the frontline and confronted with the death of their patients on an almost daily basis, adding staff is an imperative for an adequate activity, which highlights the issue of mobility management. Hence, COVID-19 mobility is related to caregivers faced with unfamiliar working conditions, voluntarily or involuntarily, for unknown periods (several days to several weeks), and caring for patients with life-threatening conditions.

Although implementing occasional mobility in this context is a likely cause of exhaustion, professional stress, and absenteeism, typically, these aspects have been largely ignored by necessity during the COVID-19 pandemic, to let emerge a new and flexible organization among departments and allow receiving patients in a massive and urgent manner [2]. The above-described situation gives rise to two phenomena that have not been addressed with equivalent level of attention: first, the comparison of frontline and second-line COVID-19 healthcare workers [3] has shown higher anxiety and depression levels [4] associated with deteriorated sleep quality and long-term post-traumatic stress in frontline workers [5]; second, the densification of adequate management of the mobility has deeply impacted the mission of human resources teams, with possible consequences on their health. Although managers are aware of mobility-associated drawbacks, they had to deal with both an increased workload and complicated decision making, which does not exempt them from occupational stress.

In addition to significant adjustments of workload and work style due to mobility, caregivers and managers have had to reorganize their family life. The mistrust of their entourage toward their risky job, the fear of spreading the virus outside the hospital, and the distress about infecting their own family [6,7] represent common daily life difficulties [2]. As an illustration, a significant proportion (about 20%) of frontline staff reported the need for advice from specialized professionals (psychologists in particular) and for greater support from their managers [8]. Not surprisingly due to this combination of difficulties, the first wave has fostered a state of stress [7,9]. The arrival of the second wave and the start of the vaccination campaign have subsequently forced hospitals to build a new plan grounded on a degraded situation. So, in a preponderant way at this stage of the crisis, there was a need to apprehend individual stress profiles among staff affected by mobility.

Stress is considered one of the most common health risks that accounts for more than 10% of all work-related illness claims [10]. Stress was first defined in the 1970s as “the non-specific response of the body to any demand upon it” based on hormonal dynamics [11]. In the following years, psychological stressors have been progressively identified as they relate to how we interpret situations or events, which modulates the organic response. It follows that stress is rather a highly individual experience based on the transaction between an individual and its environment, as suggested in the widely used transactional model described by Lazarus and Folkmans [12].

Occupational stress can be defined as a set of biological, psychological, and social responses to work-related stressors. Importantly, the same stressful situation may not have the same consequences from one individual to another; dispositional factors including e.g., personality, anxiety trait, life experiences, and mood combined with environmental factors that become preponderant in case of occupational mobility, can modulate the capacity of an individual to perceive and evaluate the current imbalance [12]. Prolonged exposure to occupational stressors associated with the inability to implement adequate responses to stressors can lead to resource depletion and is a major cause of chronic disease. Coping is a process that has received an evolutive definition but in essence describes one’s reaction to manage a particular demand that is deemed taxing or overwhelming [13]. Core concepts emerging in coping range from problem-focused to emotion-focused or active coping to avoidance coping. When adopting active coping, a person makes effort to change (actively) a stressful situation while avoidance relies on activities or mental states that help one to stay away from or escape a stressful situation. It is generally admitted that active coping is more efficient for facing a stressing situation while avoidance coping is identified the risk factor [14]. The elaboration of coping questionnaires has a long history. A number of coping questionnaires are available to assess the coping strategies adopted by various people, to name a few, the COPE inventory or its abbreviated version. Both contain interesting subscales to grasp problem-focused coping strategies as well as emotion-focused coping strategies [15,16].

In the hospital setting, burnout and occupational stress have been reported as a result of exposure to work-related stressors that are experienced to varying degrees by caregivers [17]. There is recent evidence that COVID-19 can exacerbate work-related stressors at hospitals with reports pointing to stress depending on gender [18], seniority [18], nursing occupation [19], rejection [18,20], and lack of social support [21,22]. A degraded psychological status has been reported in healthcare workers [4,23], with high levels of anxiety and depression measured in caregivers [24,25,26], which are associated with insomnia and feelings of fear of contracting or transmitting the virus [27]. Ultimately, cases of suicide have been observed [2]. The stress generated by the pandemic crisis likely persists in the form of post-traumatic stress [28,29], which is a scenario already observed in past epidemics [30]. Taken together, these observations reinforce the importance of health monitoring, support, and assessment in healthcare personnel [18].

Monitoring stress has found reliable support in obtaining self-reported feelings through guiding questionnaires on one hand and in tracking organic markers associated e.g., to the activity of the autonomic nervous system on the other hand [31]. Highly sensitive markers have been obtained from analyses of heart rate variability, because heart rate fluctuations are under the tight control of vagal and sympathetic modulations of the sinus rhythm [32]. The link between vagal modulations of the sinus node and a well-identified central circuitry including the cortical prefrontal region, the amygdala, the cingulate cortex, and the insula represents a strong background to infer stress-related hypoactivation of the prefrontal cortex and hippocampus and hyperactivity of the amygdala [33]. By capitalizing on dynamic models of heart–brain interactions [34,35,36] and the polyvagal theory [37], an abundant literature has shown that blunted vagal modulations of heart rate at rest are associated with anxiety disorders and symptoms of anxiety such as stress, worry, and depression [38]. Interestingly, the vagal activity is reflected in short-term (high-frequency) fluctuations of heart rate (HR) [32], thus enabling researchers to access vagal markers non-invasively. For such analyses, attention has been paid to the Root Mean Square of Successive Differences (RMSSD) between normal-to-normal R-R Intervals (Heart Rate Variability, HRV) or power in high frequencies of HRV extracted from mode decomposition. The dynamical system theory provides additional markers related to nonlinear dynamics in heart rate variability, which has shown considerable appeal when it comes to explore mood [39], cognitive workload, and stress [40,41]. The focus has been mainly on the level of irregularity in HRV, which is quantified by computations of the sample entropy at several observational scales of the HRV signal, to reflect nonlinear dynamics in the above-described neurovisceral system.

The main hypothesis of the present work was that stressful situations generated by COVID-19 mobility affect mobilized caregivers and human resources staff in very specific ways. Comparatively, psychophysiological status in non-mobilized caregivers and in a representative population outside the hospital (university researchers) should be less degraded. To get a picture of these more or less marked and specific alterations, the objective was to extract relevant markers of both psychological and physiological individual status. We based our analysis on a 15-min procedure at the workplace including questionnaires and recordings of heart rate fluctuations (HRV) in quiet conditions. Classical, multivariate, and clustering analyses helped grasping population-specific impact of COVID-19.

## 2. Materials and Methods

### 2.1. Participants

The Mobil Stress project is a descriptive exploratory pilot study at Centre Hospitalier Universitaire de Bordeaux of an open cohort recruited on a voluntary basis of 16 healthcare professionals in mobility (MOB) during the second COVID-19 pandemic wave. None of the people (see below) involved in the present study have declared heart rhythm disorders or treatments involving cardiac or psychotropic drugs, which were exclusion criteria. At the moment of the experimentation, those people identified as group MOB in the present study were all experiencing COVID-19-induced mobility a in care unit, where they carry out their daily work since a few days to several weeks. Over the same period, fifteen other caregivers not experiencing mobility during the last month were recruited to form the group no-mobility (N-MOB). Fourteen people pertaining to the human resources team of administrative staff (ADM) in charge of mobility are group ADM. Outside the hospital, fourteen researchers/teachers (RES) employed by the University of Bordeaux, France are the group (RES). The main characteristics of these four groups are indicated in Table 1.

### 2.2. Protocol

All the participants taking part in the present study gave their written informed consent to data collection and data analyses (approved number ID-RCB: 2021-A00368-33). The experimental procedure lasted 15 min and was administrated at the workplace by the same unique experimenter in all groups. After putting a chest belt with bipolar electrodes (Polar H10, Polar Electro France S.A.S) equipped with a transmitter for HRV measurements, each participant was comfortably seated in front of a computer breathing at a spontaneous rate and used the mouse/keyboard to fill out psychological and sociodemographic and lifestyle questionnaires. 

The procedure offered a number of advantages to obtain reliable HRV recordings while people were filling out the questionnaires. Filling a questionnaire at the workplace is a quiet mental activity that could advantageously replace the situation currently called ‘rest’ in controlled experiments. Resting in awake people is indeed an ambiguous concept because in absence of mental demand there may be possibly exacerbating rumination on bad/happy thoughts, which represents unwanted and varying states that may mystify a ‘standard’ HRV. As an illustration, a common way to avoid such background noise has been to record HRV in people watching a neutral movie [36]. Hence, the present procedure aimed at evaluating a psychophysiological status through HRV markers that could be reliably obtained at the workplace thanks to concomitantly filling questionnaires. Furthermore, under the developed procedure, concurrent recordings of HRV and self-reported feelings offer the added advantage of combining psychological and physiological measures to elaborate individual psychophysiological status (see Factor Analysis and Hierarchical Clustering in paragraph ‘statistical analysis’), which has not been always possible in previous studies.

### 2.3. Psychological and Sociodemographic Characteristics

First, participants provided information about their sociodemographic characteristics and their lifestyle (Table 1). Then, they answered two psychological questionnaires:

- The Hospital Anxiety and Depression Scale (HADS) allows identifying anxiety disorders and depression as shown among patients in the general medical population [42]. The purpose of this test is not to discriminate between the various types of depression or anxiety states but to identify the presence of anxiety symptoms and to evaluate their severity. The questionnaire was divided into an Anxiety subscale (HADS-A) and a Depression subscale (HADS-D), both containing seven intermingled items. To screen for anxiety and depressive symptomatology, the following interpretation was proposed for each of the scores (HADS-A and HADS-D): 7 or less: no symptomatology, 8 to 10: doubtful symptomatology, and 11 and more: definite symptomatology. In the context of the present study, the French validated version was used [43]. This version was developed on an internal medicine service with 100 consecutive inpatients and validated through comparisons with other validated self-assessments.
- The Brief COPE (Brief Coping Orientation to problems experienced), an abridged version of the COPE inventory [15], is a multidimensional measure of coping strategies that presents fourteen scales: (1) active coping, (2) planning, (3) using instrumental support, (4) using emotional support, (5) venting, (6) behavioral disengagement, (7) self-distraction, (8) self-blame, (9) positive reframing, (10) humor, (11) denial, (12) acceptance, (13) religion, and (14) substance use, each of them containing two items. It is acknowledged that the scores obtained by assessing so many strategies are difficult to synthesize into specific results in quantitative analyses [16]. For this reason, we employed a recently validated procedure that is more synthetic and therefore more suitable in occupational contexts [14]. Based on a cohort of 398 individuals (cancer patients and their caregivers), the authors structured the Brief COPE by using principal component factor analysis. Validity was confirmed by exploring relationships between scores of the Brief COPE and scores of other scales such as SF36. Additionally, replication of the model was tested and showed a good fit on the two subsample groups (patients and caregivers). The authors labeled four types of coping they identified as follows: positive thinking (three strategies: positive reinterpretation, humor, and acceptance); problem solving (two strategies: active coping and planning); social support (four strategies: expressing feelings, seeking instrumental and emotional social support, religion, and spirituality); and avoidance (five strategies: behavioral disengagement, self-distraction, self-blame, denial, and substance use). The Brief COPE provides scores in the range 0 to 5, where a high score indicates great use of coping strategy. Here, we focused on the situational scale coping state where participants reported their usual way of responding to stressful situations.

### 2.4. Physiological Characteristics: Analyses of Heart Rate Variability

Heart rate variability (HRV) was assessed by a series of successive cardiac interbeat (RR) intervals duration, obtained thanks to 1 kHz sampling of electrical cardiac activity by the belt Polar H10. The transmitter was connected via Bluetooth to an android smartphone, and successive intervals delivered by the transmitter were recorded and stored in the smartphone. The Polar device has the capacity to record the heart electrophysiological signal associated to ventricular contraction (R-peak) with a quality similar to ECG [44,45]. Individual RR time series were imported to Matlab (Matlab Release 2021a, Mathworks, Natick, MA, USA) for subsequent analyses using both existing functions and custom-designed algorithms. The raw data were inspected for artifacts. Occasional ectopic beats (irregularity of the heart rhythm involving extra or skipped heartbeats such as extrasystole and consecutive compensatory pause) were visually identified and manually replaced with interpolated adjacent RR interval values.

#### 2.4.1. Time-Domain and Frequency-Domain Analyses

In time-domain, the mean RR interval and the root mean square of successive differences (RMSSD) were computed. RMSSD relies on magnitude in short-range (due to differentiation) fluctuations of heart rate and is generally associated to vagal tone modulation [32]. A quite similar information is generally derived from power in the high-frequency domain computed from power spectral density (PSD) of RR series. Here, a discrete Fourier transform was performed after 4 Hz resampling of RR intervals using a cubic spline interpolation. The HRV power in two fixed bands 0.04–0.15 Hz and 0.15–0.4 Hz called LF and HF were computed as well as the ratio LF/HF, which is a classical index of the sympathovagal balance.

#### 2.4.2. Entropy in HRV

Complex regulation mechanisms in biological systems have been explored by assessing the level of irregularity in signal output, which is reflected e.g., in the sample entropy profile over a range of observational scales [46]. I have been advised that marked trends in the signal can affect the evaluation of the standard deviation, which is a critical parameter for entropy computations [47]. Therefore, HRV series were first pre-processed to remove potential trends. For that, empirical mode decomposition, a data-driven method, was used to decompose HRV series into a sum of intrinsic mode functions (IMFs) and a residual trend. The residual trend was removed.

Refined composite multiscale entropy (RCMSE) based on the algorithm proposed by Wu [48] was computed on a detrended series. Using the RCMSE algorithm is particularly advised when applying multiscale entropy methods to short lasting series. The RCMSE approach addresses most of the issues associated to other multiscale methods [48].

RCMSE was computed as:

(1) $$\mathrm{RCMSE}\left(x,\tau ,m,r\right)=-\ln\frac{\sum_{k=1}^{\tau }n\frac{m+1}{k,\tau }}{\sum_{k=1}^{\tau }n\frac{m}{k,\tau }}$$

where *τ* is the scale factor, m is the embedded dimension (here *m* = 2), and *r* is the tolerance factor set here to the fixed value of 0.15. The RCMSE value at a scale factor *τ* is defined as the natural logarithm ratio between the mean of the number of *m* dimensional matched vector pairs and is constructed from the *k*-th coarse-grained time series at a scale factor of *τ*. The proportion of the same vectors characterizes the level of signal regularity, which is an indicator of poor signal complexity. Since signal length uniformization is critical for reliable entropy determination, the length of each analyzed series was determined based on the shortest series among an individual set of recordings. As a consequence of the series length, RCMSE was assessed over a range of scales from 1 to 5 to preserve >150 samples per scale. An entropy index, Ei was used to characterize entropy in a series by a single marker; Ei evaluated the area under the curve of RCMSE vs. scales obtained by using the trapezoidal rule [49,50].

#### 2.4.3. Statistical Analysis

Throughout the document, values are expressed as mean and standard deviation to the mean (SD). Outliers were identified using the ROUT method [51].

The group sizes were determined by power calculation (GPower version 3.1.9.6, https://www.psychologie.hhu.de/arbeitsgruppen/allgemeine-psychologie-und-arbeitspsychologie/gpower, accessed on 2 August 2021) based on our preliminary data [40,50] and obtained during pre-testing on caregivers (α error probability: 0.05, power 0.8) mean RMSSD value, 24 ms; standard deviation, 12 ms; mean difference, 9%. This resulted in n = 17 for one experimental group (Cohen’s d effect size: 1.18).

Inter-group differences (MOB, N-MOB, ADM, RES) in main variables, HADS scores, coping strategies scores, and cardiac markers were evaluated by repeated measures analysis of variance (ANOVA) and post hoc Tukey correction. All the variables satisfied the conditions of normality, which were tested with the Shapiro–Wilk test and Levene test (homogeneity of variance in samples).

A value of *p* < 0.05 was considered to indicate statistical significance.

Factor Analyses for Mixed Data (PCAmix), which combined principal component analysis (PCA) and multiple correspondence analysis (MCA), were performed taking into account the presence of both qualitative (groups) and quantitative data. This method allows reducing data to identify proximity between variables and proximity between observations. Subsequently, a Hierarchical Clustering on Principal Components (HCPC) was performed to create homogeneous clusters of subjects based on quantitative data.

Bayesian equivalents to classical tests were used to extend insight of the significance (*p*-values), according to the likelihood of the alternative hypothesis versus the null hypothesis. Specifically, the log scale of BF10 (Bayes factor giving evidence for the alternative hypothesis H1 over the null hypothesis H0) noted log(BF10) was calculated. Negative values of log(BF10) support for the null hypothesis, whereas positive values support the alternative hypothesis (see [52] for an interpretation scale of log(BF10)).

Statistical analyses were performed on RStudio (Version 1.2.5033, RStudio Team (2019). RStudio: Integrated Development for R. RStudio, Inc., Boston, MA URL http://www.rstudio.com/, accessed on 4 September 2019) to compute ANOVA, Factor Analyses for Mixed Data (PCAmix), and Hierarchical Clustering on Principal Components (HCPC), and on JASP (Version 0.14.1, https://jasp-stats.org/, accessed on 30 August 2021) to compute log(BF10).

## 3. Results

### 3.1. Hospital Anxiety and Depression Scale (HADS)

**Figure 1:**
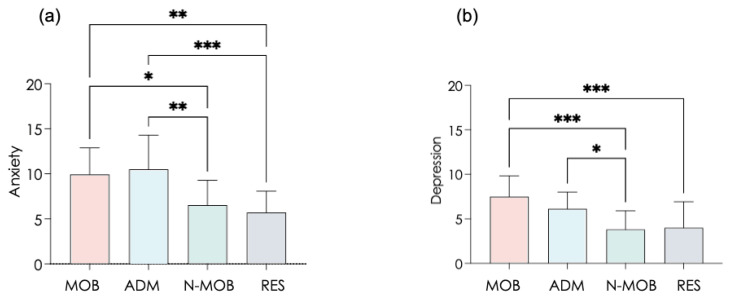
Scores of Anxiety subscale (**a**) and Depression subscale (**b**) calculated from the Hospital Anxiety and Depression Scale (HADS) in each tested group. MOB, mobilized healthcare workers; ADM, administrative staff; N-MOB, healthcare professionals who had not been mobilized; and RES, researchers and teachers from the University. Error bars represent the standard deviation. * *p* < 0.05; ** *p* < 0.01; *** *p* < 0.001.

As a main observation here, anxiety scores significantly differed among the tested populations, reaching the highest values in MOB and in ADM (F(3,55) = 9.24, *p* < 0.0001, log(BF10) = 6.19) (Figure 1a). In more detail, eight out of 16 MOB and six out of 14 ADM reached scores ≥11, which served as a reference to infer symptomatic anxiety in the literature. On average, anxiety scores in other populations (N-MOB and RES) were only half as high. 

A similar inter-group profile was observed regarding depression (F(3,55)=8.75, *p* < 0.0001, log(BF10) = 5.83) (Figure 1b) with yet less symptomatic subjects. Although high anxiety scores in MOB was an expected observation, quite equivalent scores in ADM is a salient observation. In comparison, N-MOB (incidentally RES) were less affected, thus shedding light on the specific degradation in staff directly concerned by mobility.

### 3.2. Brief Cope

**Figure 2:**
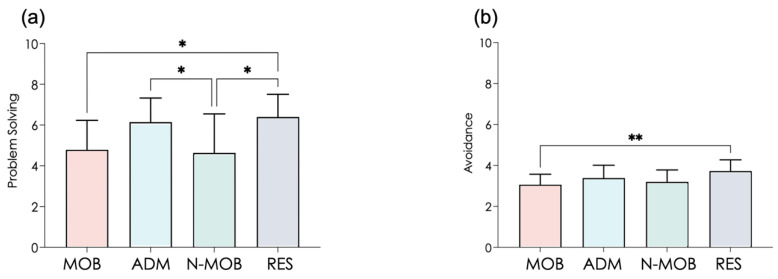
Scores on the two factors of the Brief-COPE variables significantly different between subjects (see Table 2), Problem Solving (**a**) and Avoidance (**b**). MOB, mobilized healthcare workers; ADM, administrative staff; N-MOB, healthcare professionals who had not been mobilized; and RES, researchers and teachers from the University. Error bars represent the standard deviation. * *p* < 0.05; ** *p* < 0.01.

Among the multiple dimensions of brief coping, only problem solving and avoidance reached significant difference among groups (Table 2 and Figure 2). In contrast with anxiety results, both groups of caregivers (MOB and N-MOB) exhibited degraded scores in problem solving. ADM was less affected than caregivers, since ADM reached scores similar to RES. Again, inter-group divergence in anxiety and problem-solving scores points to group-specific profiles, which were more finely analyzed in subsequent clustering analyses.

### 3.3. Cardiac Autonomic Markers

**Figure 3:**
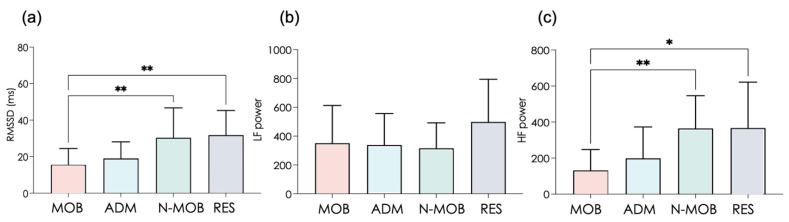
Time and frequency domains markers extracted from RR intervals time series. (**a**) Root Mean Square of the Successive Differences, RMSSD; (**b**) power in low frequencies, LF power; (**c**) power in high frequencies, HF power. Error bars represent the standard deviation. * *p* < 0.05; ** *p* < 0.01.

Caregivers in mobility (MOB) exhibited the lowest heart rate variability in short-range dynamics (RMSSD and HF power, Figure 3a,b). Interestingly, when comparing MOB to N-MOB, RMSSD (F(3,55) = 6.45, *p* < 0.001, log(BF10) = 3.77) and HF power (F(3,55) = 6.17, *p* < 0.01, log(BF10) = 3.52) were much more degraded in MOB, thus indicating that mobility per se affects vagally-mediated parasympathetic modulations among caregivers (Figure 3a). The autonomic impairment was specifically reflected in the vagal branch of the autonomous nervous system, since sympathetic control reflected in LF power (Figure 3c) was unaffected (F(3,55) = 6.17, *ns*). The latter is confirmed by the negative value of log(BF10) (−2.29) that indicates a very strong evidence for the null hypothesis (H_0_).

**Figure 4:**
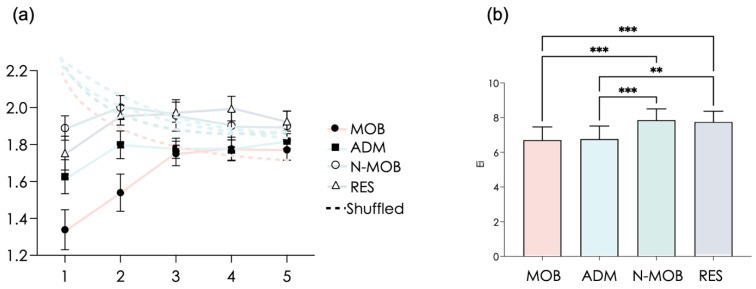
Refined composite multiscale entropy (RCMSE) analysis of RR interval time series. (**a**) Sample entropy values at time scales 1 to 5 are reported for each group (MOB, ADM, N-MOB, RES) (mean ± SD). The RCMSE curves for the surrogate shuffled time series are also presented (dotted lines). The entropy index represents the trapezoid approximation of the area under each curve; (**b**) mean values of the entropy index (Ei, arbitrary unit). ** *p* < 0.01; *** *p* < 0.001.

Nonlinear dynamics of HRV made a clear distinction among groups, which is an important observation that resembles what has been obtained with anxiety scores (Figure 4). The superiority in HRV-entropy (RCMSE) over other HRV markers is illustrated by log(BF10) values that overperformed (see below) those obtained with RMSSD and HF power (see above). The lowest values of RCMSE were observed in MOB and ADM with a Bayes factor >8 (F(3,55) = 11.53, *p* < 0.0001, log(BF10) = 8.13). When compared to N-MOB and RES, people affected by mobility (MOB and ADM) showed much degraded complexity in HRV dynamics. This is not trivial when it comes to interpreting neurovisceral correlates of vagal and nonlinear markers.

### 3.4. Factor Analyses for Mixed Data (PCAmix)

**Figure 5:**
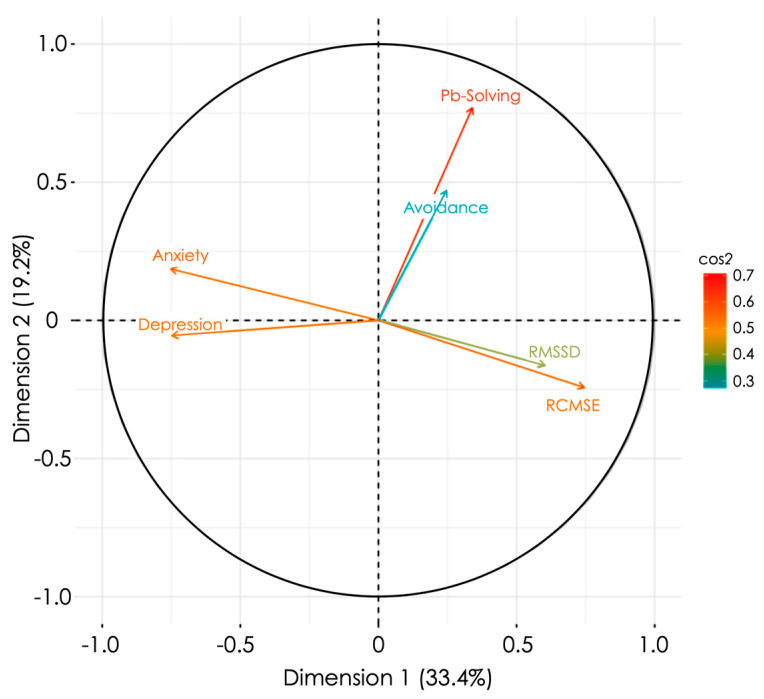
Principal component analysis of mixed data with the two main dimensions projected on orthogonal axes. Anxiety and Depression scores were calculated from the Hospital Anxiety and Depression Scale (HADS). Problem solving (Pb-Solving) and avoidance represent the two Brief-COPE subscales that reached significant difference among the tested populations. RMSSD represent the heart rate variability in short-range dynamics, and RCMSE represents the complexity in nonlinear dynamics of heart rate variability. Only quantitative variables are represented in the figure.

**Figure 6:**
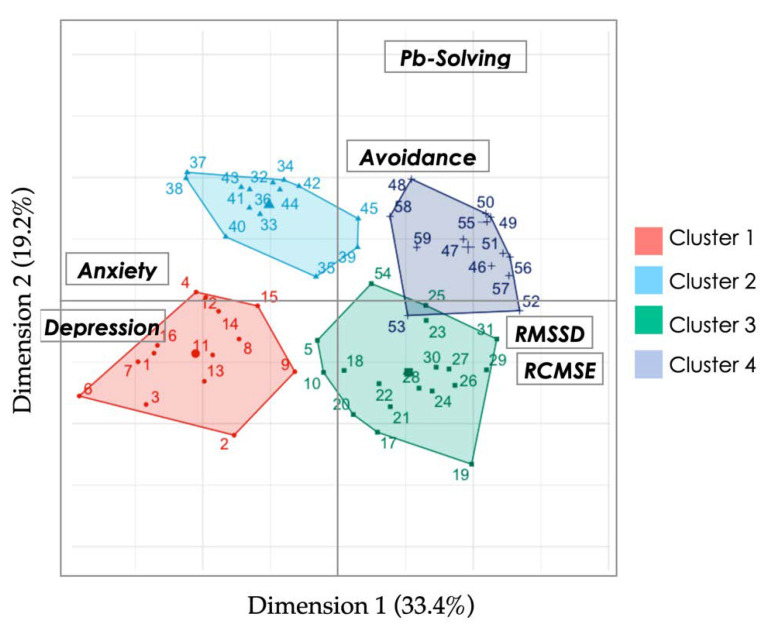
Mapping of the clusters as a function of PCAmix axes: Dim1 *x*-axis and Dim2 *y*-axis are defined from results illustrated in Figure 5. Individuals are numbered and colored according to their cluster. The barycenter of each cluster is represented by a larger symbol.

The whole set of reliable markers identified by the above statistical analyses was submitted to a hierarchical clustering on principal component analysis, which comprises two steps. First, a factor analysis for mixed data (PCAmix) was performed to disclose covariations among variables and select the two main PCA dimensions (Figure 5). Second, a hierarchical clustering on the two principal components was performed to cluster people with similar characteristics (Figure 6). The two retained dimensions explained respectively 33.4% and 19.2% of the total variance. Anxiety (18.8%), depression (18.6%), and HRV entropy (RCMSE, 11.4%) were the most salient markers to describe Dim1. Problem solving (a facet of coping) best described Dim2 (43.4%). The orthogonality between anxiety/depression/entropy (Dim1) and coping (Dim2) indicates the absence of covariation between these different facets of individual psychophysiological status.

### 3.5. Hierarchical Clustering on Principal Components (HCPC)

HCPC was performed subsequently to PCAmix and clearly provided four clusters having similar characteristics (Figure 6). Each participant is positioned within the cluster he belongs to, according to Dim1 (Anxiety/Depression/entropy) and Dim2 (Problem solving). Interestingly, the clustering output by the HCPC matched perfectly with the distinctive populations we tested in the present study. Cluster 1 includes MOB and was mostly characterized by marked depression, degraded entropy (RCMSE), and vagal (RMSSD) markers. People pertaining to ADM were in cluster 2 defined by high anxiety and degraded entropy (RCMSE). N-MOB was identified in the cluster 3, which was characterized by maintained entropy and RMSSD, the absence of anxiety/depression but blunted coping strategies. RES matched with cluster 4, exhibiting the highest scores in coping and lowest scores in anxiety/depression.

All in all, caregivers all exhibit degraded capacity in problem solving (when compared to ADM and RES) but distinguish themselves (MOB vs. N-MOB) by levels of anxiety/depression and cardiac autonomic control (most altered in MOB). Comparatively, ADM retained intact problem-solving capacity despite obvious anxiety.

### 3.6. Multivariate Linear Regression

While considering all the participants together, performing a multivariate linear regression using backward data entry showed that overall, RCMSE, problem solving, and RMSSD significantly predict Anxiety (F(3,55) = 5.79, *p* = 0.00519):

Anxiety = 19.88 + (−1.07 × RCMSE) + (−0.41 × Problem solving) + (−0.07 × RMSSD).

## 4. Discussion

Due to the unprecedented increase in stressors and workload in the hospital during peaks of the COVID-19 pandemic, there is a pressing need to assess the expected deterioration in the psychophysiological status of staff whose lives are affected by occupational mobility. Based on a simple experimental setup that consisted in 15-min testing in a quiet environment at the workplace that included questionnaires and concomitant HRV recordings, the present study provides four main findings:

(i) Caregivers in mobility (MOB), and more surprisingly human resources people (ADM), demonstrated high levels of anxiety, which was effectively reflected in a degraded cardiac autonomic control;
(ii) Comparatively, less degraded psychophysiological status was observed in non-mobile caregivers (N-MOB) and university researchers (RES), despite blunted coping strategy in N-MOB;
(iii) Five markers were prevalent to establish distinctive psychophysiological profiles, namely anxiety, depression, problem solving, vagally-mediated HR and complexity in cardiac autonomic control;
(iv) Among these markers, HR complexity (assessed by multiscale entropy) and problem solving appeared to be highly relevant for the exploration of crisis-induced anxiety.

An obvious amplification of occupational stress has been reported during the COVID-19 pandemic in care workers in association with psychological distress [4,23], depression, anxiety, insomnia [24,25,27], and some cases of suicide [2]. Here, we show that this amplification is linked to organic stress as shown by low levels of vagal autonomous modulation of the heart rhythm measured in quiet conditions in care workers that are at the frontline during the second peak of the crisis.

Assessing anxiety and depression through the Hospital Anxiety and Depression Scale (HADS) showed that MOB and ADM presented significantly higher values than N-MOB and RES in the both HADS-A and HADS-D scales. Previous research showed that scores between 0 and 7 can be considered normal, so that with a score of 11 or more in ±50% of MOB and ±45% of ADM (Figure 1), anxiety obviously reached high levels in these two groups. The similar level of anxiety observed in ADM is a striking result with obvious consequences for work organization during crises. Self-reported levels of anxiety matched finely with physiological cues about cardiovascular control extracted from HRV, e.g., RMMSD, which is a marker that is easy to get in a few minutes.

RMSSD is a widely used marker of vagal autonomous behavior in resting conditions, whose value depends on age and psychophysiological health [53]. Quantifying RMSSD directly informs on the short-term modulation of HRV by the parasympathetic activity, which is a transdiagnostic marker of psychopathology [54], self-regulation [55], and cognitive control [56]. By compiling HRV recordings across about 10,000 working adults, Jarczok MN et al. [53] reported that daytime (awake) values of RMSSD below 25 ± 4 msec indicate an elevated risk across a range of cardiovascular risk factors. Remarkably, RMSSD values below this cut-off were observed here in MOB and ADM but not in N-MOB and RES. It means that staff most directly affected by mobility in the present study, MOB and ADM, presented high risks of health-related defection or putative professional mistakes in times of intense mobilization at the hospital. Here, markers such as anxiety, depression, and RMSSD made a clear distinction between MOB/ADM and N-MOB/RES (Figure 1 and Figure 3).

Recent intersections between dynamical systems theory and the behavior of physiological system have shown that there is much to learn from the temporal organization of systems dynamics, which is assessed by complexity metrics. Capitalizing on advances in nonlinear methods, Young and Benton showed that mood and integrated functioning of the brain are well captured by HR complexity reflected in entropy markers [39]. RCMSE used in the present study is one of the most reliable algorithm for exploring complexity in short HRV series [48]. As reflected here in a number of statistical evidence (Bayes factor, ACP, clustering, multivariate linear regression), HR entropy is critical in analyzing psychophysiological profiles. Psychology and neurosciences have associated HR complexity with specific activity in neural networks embedded in high-level instances of the brain and connected to the cardiovascular control centers in the brain stem. A number of experimental studies point to a critical role of nonlinear interactions governing instances in the Central Autonomic Network and a specific role played by the amygdala [50,57,58]. Critical functions arise from this network coordination. Flexibility and goal-directed behavior rely on adequate connection between the prefrontal cortex and the heart through the CAN, which have been evidenced by neuroimaging [57,58]. HR complexity is believed to reflect dynamical coordination in brain-to-heart interactions [40,50,52], which are essential for adaptivity. Anxiety is known to impair the recruitment of prefrontal control mechanisms and to disrupt the circuitry of the CAN, thus leading to an amygdaloid hyper-responsivity [59]. When the context is perceived as stressful, the circuitry linking the prefrontal cortex and the amygdala, a component of the CAN and a target of anxiety, plays a key role in a degraded behavioral and physiological dysregulations [40,60,61]. Recently, in specifically designed experimental setups, a degraded complexity (decrease in RCMSE) has been associated to a sudden rise in induced anxiety [40]. In the same vein, anxiety and RCMSE covaried in workers self-reporting stress at work [50]. Entropy assessed by RCMSE was also the most sensitive marker to show stress contamination in dyads [62]. Lastly, when it comes to mobilizing specific executive functions, RCMSE is the most sensitive marker of cognitive–autonomic interactions [52]. Taken together, above results lead to concluding that the strong degradation in HR complexity in staff confronted to mobility in the present study likely signals a markedly degraded psychophysiological status that could be put in relation with a severe dysfunction of the CAN during a pandemic peak in MOB and in ADM. As CAN plays multiple roles in organic health, cognition, and behavior, RCMSE likely provides a reliable index for assessing the level of attention that should be given on individual workers to prevent severe, multisystem degradation over time. In response to CAN degradation, an efficient countermeasure could be cardiac coherence training, which consists in enhancing bottom–up (heart-to-brain) modulations thanks to breathing at a rate matching the Mayer waves (0.1 Hz). Short periods (5 min) twice a day of coherence training are sufficient to restore neurovisceral complexity and stress defense in moderately stressed workers reporting difficulties for balancing work, family, and lifestyle [50].

Research in psychology shows that if a person thinks that a given situation is out of hand, this person will likely turn away from the problem without trying to cope. Thus, how the same situation is perceived by different people can influence individual coping strategies. When one is centered on the problem, the situation is less stressful, but focusing on emotions exacerbates dramatization. As expected in the present study, MOB presented altered scores of problem solving and avoidance, which are two of the four labeled factors of the Brief-COPE test. In that, they were similar to N-MOB, although the later showed less anxiety. ADM exhibited high stress levels but maintained coping strategies. Thus, perceived stress per se can hardly be incriminated in blunted coping. Interestingly, the combination of psychophysiological markers used in the present study makes it possible to discriminate between people with alteration in problem solving, which concerned caregivers in general (MOB and N-MOB), people with marked anxiety, which concerned MOB and ADM, and specific profiles combining both drawbacks thanks to clustering (Figure 6). The above results deserve two important remarks. First, the information that ADM retained their capacity of problem solving despite obvious anxiety during crisis peaks has great value for future crises management, because much of the success in healthcare systems is based on adequate management for which the decision-makers must be resolute under stressful conditions. Second, the question that arises is why ADM but not MOB can continue to solve problems when both groups exhibit the same dramatically high level of anxiety. The main intuition is that these populations may not share the same contamination threat, since MOB (but not ADM) are in contact with COVID patients, exacerbating the fear of spreading the virus upon returning home and the needed protective measures [4]. Therefore, in times of crisis, time spent outside the hospital may no longer provide a context for positive emotions (hugging loved ones, etc.), yet it is crucial for mental health [63]. Effective countermeasures could be based on any method that isolates people from ongoing stressors while stimulating positive emotions. Virtual reality tools hold promises for providing such immersion.

The clustering analysis demonstrated clear group-specific profiles (Figure 6), thereby facilitating psychological and physiological markers interpretations. MOB (cluster 1, Figure 6) combined high anxiety and degraded capacity of problem solving in association with blunted vagal activity, and neurovisceral dysfunctions reflected in HR complexity. ADM (cluster 2) despite anxiety and degraded neurovisceral functions retained their capacity for problem solving. In comparison, N-MOB developed no obvious anxiety, which matched with intact neurovisceral functions (HR complexity). The group that served as control (RES, cluster 4) exhibited poor anxiety during the crisis when compared to hospital staff in association with good prognostic psychophysiological markers.

What is unveiled by the clustering analysis is an obvious interest in differentiating psychophysiological status to adapt countermeasures. Therefore, remediation techniques and other types of coping management training will need to be offered differently depending on the target population. In short, VR focused on positive emotions and stress management could be preferred for MOB but cardiac coherence could be preferred for ADM. However, the clustering analysis also identifies the more marked degradations that should be expected in times of crises, thus guiding prevention methods to be used individually in anticipation of future crisis. To take care of caregivers who will be confronted with a mobility in the future, prevention could efficiently include detection of weak signals of psychophysiological degradation. In this perspective, a recent study on healthcare workers presents preliminary results supporting the possible identification of COVID-19 symptoms from daily HRV monitoring [64]. Our multivariate analysis may point to the predictive ability of HR complexity to anticipate subtle alterations of the CAN as a source of systemic health degradation [65,66]

## 5. Conclusions

In conclusion, the results of the present study show that during a COVID-19 pandemic peak, occupational stressors shape fine individual psychopathological status, which is probably due to the intense upheaval in occupational stress. Anxiety demonstrated clearly distinct levels and is known to have a multitude of mental and organic functional targets and origins. The multiple linear regression provided the weight of explanatory variables to quantify individual levels of anxiety, which shed light on two main markers: HR complexity and in a lesser extent the coping strategy problem solving. When combined together, these markers draw a very specific profile that may serve as a tool for individualized countermeasures to prevent health and occupational risks.

Obtaining exploitable data in times of crisis in overworked caregivers is a challenge. We show that a 15-min procedure at the workplace can provide relevant variables to account for finely shaped psychophysiological profiles. During this procedure, maintaining a mental charge through questionnaires while recording HRV has certainly been an asset, since HRV markers helped clearly distinguish each group, where the absence of mental focus could have provided heterogeneity. Despite relative homogenous results, groups with higher samples would certainly strengthen the present outcomes, specifically the capacity to study male and female profiles separately [67] for more even more efficient individualized strategies.

All in all, the present procedure offers a way to detect specificity in psychophysiological profiles, which is an asset to guide individual strategies in both remediation and prevention domains.

## Figures and Tables

**Table 1:** Sociodemographic and lifestyle characteristics of the population.

	MOB n = 16	N-MOB n = 15	ADM n = 14	RES n = 14
Age (years)	41.87 ± 10.05	35.80 ± 13.09	44.29 ± 14.07	39.92 ± 12.91
Gender				
Female	n = 14	n = 14	n = 13	n = 6
Male	n = 2	n = 1	n = 1	n = 8
Marital status				
Married or in a relationship	n = 14	n = 8	n = 9	n = 10
Single	n = 2	n = 7	n = 5	n = 4
Professional seniority (years)	12.30 ± 8.36	9.10 ± 11.28	8.21 ± 6.66	10.67 ± 9.47
Education level				
Bachelor’s degree	n = 6	n = 6	n = 4	n = 0
Master’s degree, Ph.D.	n = 10	n = 9	n = 10	n = 14
Smokers	n = 4	n = 1	n = 1	n = 1
BMI (kg·m^−2^)	24.34 ± 4.19	23.51 ± 3.14	28.21 ± 6.66	24.41 ± 2.14
Already had COVID-19	n = 1	n = 1	n = 0	n = 0
Physical activities (hour/week)				
≤3	n = 13	n = 10	n = 9	n = 11
>3	n = 3	n = 5	n = 5	n = 4

**Table 2:** Means and standard deviations of the Brief-COPE subscales according to the type of emotional situation.

Brief Cope Dimensions (Items)	MOB n = 16	N-MOB n = 15	ADM n = 14	RES n = 14	Statistical Significance
Positive thinking	5.25 ± 1.20	5.35 ± 1.14	5.93 ± 0.87	5.40 ± 1.00	*ns* (log(BF10) = −1.25
Problem solving	4.78 ± 1.45	4.63 ± 1.91	6.14 ± 1.18	6.39 ± 1.11	F (3,55) = 5.69; *p* = 0.002 log(BF10) = 3.05
Social support	4.00 ± 1.05	4.48 ± 1.14	4.18 ± 0.82	4.20 ± 1.09	*ns* (log(BF10) = −1.82
Avoidance	3.05 ± 0.51	3.20 ± 0.58	3.39 ± 0.62	3.73 ± 0.55	F (3,55) = 3.92; *p* = 0.01 log(BF10) = 1.40

## Data Availability

The data presented in this study are available on request from the corresponding author. The data are not publicly available due to data sensitivity.
